# Symmetry-locked six-state control of altermagnetism via sliding ferroelectricity

**DOI:** 10.1126/sciadv.aec5229

**Published:** 2026-06-03

**Authors:** Wei Sun, Wenxuan Wang, Changhong Yang, Shifeng Huang, Zhenxiang Cheng

**Affiliations:** ^1^ Shandong Provincial Key Laboratory of Green and Intelligent Building Materials, University of Jinan, Jinan 250022, China.; ^2^ School of Material Science and Engineering, University of Jinan, Jinan, Shandong 250022, China.; ^3^ Institute for Superconducting & Electronic Materials, Australian Institute of Innovative Materials, University of Wollongong, Innovation Campus, Squires Way, North Wollongong, NSW 2500, Australia.

## Abstract

Altermagnetic multiferroics offer a promising route to low-power spintronics by enabling spin splitting without net magnetization, extending beyond conventional spin-orbit coupling. However, achieving deterministic electric control has remained elusive. Here, a six-state platform for high-dimensional magnetoelectric coupling in altermagnets is established by exploiting the nondegenerate transition paths of sliding ferroelectrics as a symmetry-engineering knob, thereby transcending the conventional binary (up/down) paradigm of sliding ferroelectricity. First-principles calculations on bilayer manganese phosphorus trisulfide reveal a spin-polarization symmetry-locking mechanism. Polarization switching along the three nondegenerate paths not only reverses the spin splitting but also rotates its spin-splitting texture in 120° increments, yielding six nonvolatile, electrically addressable altermagnetic states. Furthermore, direct correspondence is established between the spin-splitting texture and the nonlinear Hall response, providing unique electrical fingerprints for each state. This work establishes a paradigm for electric field–driven reconstruction of momentum-space spin geometry, providing a versatile platform for controlling quantum phenomena in altermagnetic spintronics.

## Teaser

A symmetry-locked method for controlling altermagnetism via sliding ferroelectrics, yielding six distinct nonlinear Hall signals.

## INTRODUCTION

Multiferroic materials, which exhibit coupled ferroelectric and magnetic orders (*1*, *2*), offer a transformative platform for developing low-power spintronic devices. Among these, altermagnetic multiferroics (*3*–*6*) represent an emerging and highly promising paradigm, distinguished by several unique advantages: compensated magnetic order (eliminating stray fields), momentum-dependent spin splitting (enabling precise spin-current control) (*7*–*11*), and, crucially, an intrinsic symmetry-locked magnetoelectric coupling that surpasses the limitations of conventional spin orbit–mediated mechanisms (*12*, *13*). This coupling originates from the interplay between real-space crystal symmetry and spin configurations within the spin-space group framework (*14*, *15*), directly tying ferroelectric polarization to spin polarization in momentum space, rather than in real space. Nevertheless, despite theoretical advances, achieving deterministic electrical control over altermagnetism—particularly the reversible manipulation of spin splitting—remains an outstanding challenge.

Sliding ferroelectricity (*16*–*22*) offers a unique and promising pathway to address this challenge (*13*, *23*, *24*). Unlike conventional ferroelectrics, the polarization generated through sliding is intrinsically tied to stacking configuration, inherently breaking spatial inversion symmetry (P)—a prerequisite condition for altermagnetism. In the conventional binary picture, sliding ferroelectrics are typically regarded as hosting only two opposite polarization states. Consequently, the three nominally equivalent sliding directions along the hexagonal high-symmetry lattice vectors ([100], [010], [110]) have therefore been considered degenerate (*25*). Yet, the rich interplay between these crystallographically distinct sliding pathways and magnetic order has been largely overlooked, leaving a fundamental gap in understanding the directional selectivity of magnetoelectric coupling.

In this work, we demonstrate an approach that leverages the inherent directional anisotropy of sliding ferroelectricity to achieve deterministic, multistate control of altermagnetism in van der Waals bilayers. Using an artificially stacked bilayer of the prototypical altermagnetic MnPS_3_—high-quality monolayer and stacking-induced room-temperature stable sliding ferroelectric behavior has been experimentally confirmed (*26*, *27*), providing a robust foundation and strong motivation for theoretical studies of sliding ferroelectricity and altermagnetism in this system—we show that sliding-induced polarization serves as a multiaxial electrical switch to deterministically manipulate the spin-splitting texture. Key to this capability is the directional locking between the in-plane polarization and the crystal mirror symmetry, which imposes strict symmetry constraint on the altermagnetic order. This allows ferroelectric switching to simultaneously reverse spin channels via a pseudotime-reversal operation and rotate the spatial anisotropy of the altermagnet in 120° increments.

Our work establishes sliding ferroelectricity as a powerful tool for symmetry engineering, enabling high-dimensional magnetoelectric coupling in altermagnets. We demonstrate six nonvolatile states with distinct spin-splitting configurations, each directly controlled by an electrically switchable polarization vector. As a functional outcome of this multistate control, we observe electrically nonlinear Hall (NLH) response mediated by the Berry curvature dipole (BCD), which can be selectively switched via sliding ferroelectricity. This represents an unprecedented paradigm in magnetoelectric coupling, defined by a deterministic, symmetry-enforced coupling relationship between sliding ferroelectric states and altermagnetic spin-splitting geometry. This work opens avenues for device concepts such as multistate memory, nonbinary logic, and other spin-electronics applications requiring high-dimensional state control.

## RESULTS

**Fig. 1.**
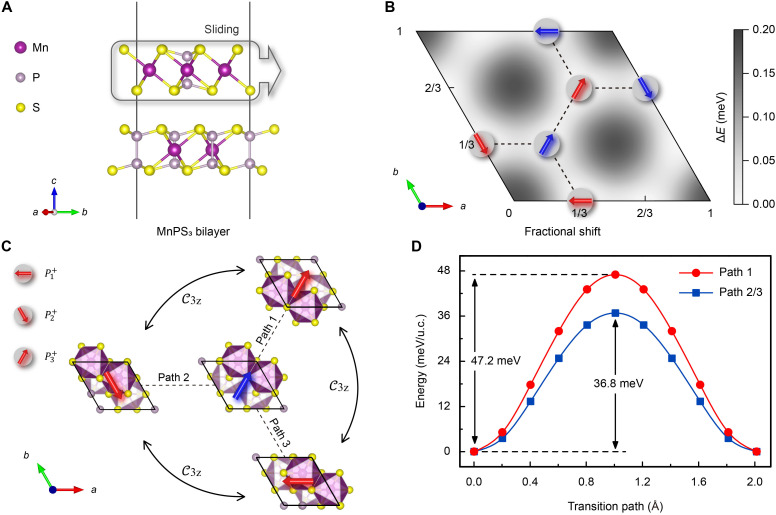
Structure and ferroelectricity of bilayer MnPS_3_. (**A**) Side view of the antiparallel stacked bilayer MnPS_3_. (**B**) Energy distribution texture for different translations, where arrows highlight the in-plane polarization direction at the energy minima, with red and blue arrows indicating the opposite out-of-plane components. (**C**) Allowed polarization transition paths and their corresponding structural symmetry relationships. (**D**) Transition energy barriers for ferroelectric switching.

By artificially stacking layers, we introduce sliding ferroelectricity into a magnetic system to construct a bilayer MnPS_3_ altermagnetic multiferroic. Before investigating the electrical control of altermagnetism, we first performed a detailed examination of the sliding ferroelectric behavior. Monolayer MnPS_3_ crystallizes in the space group $P\bar{3}1m$ and has P symmetry, and its detailed electronic and magnetic properties are shown in fig. S1. However, in the bilayer structure with antiparallel stacking, as illustrated in Fig. 1A, asymmetric interfacial coupling induces interlayer charge transfer (fig. S2), which gives rise to both in-plane and out-of-plane composite polarization. This polarization is locked by the relative lateral sliding between the layers.

Through first-principles calculations, we identified the energy minima corresponding to various stacking configurations, which are described by the translation vector of the top layer **r** = η**a** + ν**b**, illustrated in Fig. 1B. The energy maximum occurs at the fully eclipsed stacking configuration (**r** = 0). In contrast, displacing the top layer by ±1/3 along the [100], [010], or [110] directions result in six degenerate global energy minima, each accompanied by a combination of in-plane and out-of-plane polarization. To characterize these six polarization states, we adopt the notation $P_{n}^{\pm }$, where the superscript ± indicates the sign of the out-of-plane polarization component, and the subscript *n* = 1, 2, 3 denotes the in-plane polarization aligned along the [100], [010], and [110] directions, respectively.

For any given polarization state, such as the $P_{3}^{-}$, shown in Fig. 1C with an in-plane component of 0.92 μC/m and out-of-plane component of 0.26 μC/m, identifying three distinct switching paths. The corresponding energy barriers for these transitions are shown in Fig. 1D. The obtained values fall well within the typical range for ferroelectric systems, being higher than the 9-meV barrier of *h*-BN bilayer (*16*) but lower than the 66-meV barrier of In_2_Se_3_ (*28*). Coupled with the stable thermodynamic tests (fig. S3), this provides strong evidence for the experimental feasibility of the switching mechanism. Path 1 reverses only the out-of-plane component, overcoming a barrier of 47.2 meV/u.c., equivalent to a $C_{2\left[110\right]}$ rotation about the [110] axis. In contrast, paths 2 and 3 involve switching both in-plane and out-of-plane components and exhibiting a lower energy barrier of 36.8 meV/u.c. These paths correspond to combined symmetry operations $C_{2\left[110\right]}C_{3z}^{1}$ and $C_{2\left[110\right]}C_{3z}^{2}$, respectively, as shown in Fig. 1C.

Since each ferroelectric ground state corresponds to three translational directions, it is not possible to achieve stable switching between the six states solely by relying on an out-of-plane electric field. The theory of bilayer stacking ferroelectricity proposes a controllable scheme for regulation via an external electric field (*25*): For paths 2 and 3, which involve switching between in-plane and out-of-plane polarization, the mutual locking between these two polarizations is used. The scheme applies an in-plane electric field, perpendicular to the switching path and parallel to the direction of in-plane polarization change (along the [120] direction for path 2 or along the [210] direction for path 3). This approach reduces the interaction energy in the form of −**E**·**P**, providing the optimal path for switching. For path 1, which only involves out-of-plane polarization switching, the state transition can be completed by applying an out-of-plane electric field while keeping the in-plane electric field constant along the in-plane polarization direction. The relationship between the direction of the applied electric field and the ferroelectric switching path is shown in fig. S4. This sliding ferroelectric behavior leads to two key conclusions: (i) Switching the polarization necessarily reverses the out-of-plane component, and (ii) the three states sharing the same out-of-plane sign (e.g., $P_{1}^{\pm }$, $P_{2}^{\pm }$, $P_{3}^{\pm }$) are related through rotational symmetry $P_{3}^{\pm }=C_{3z}^{1}P_{1}^{\pm }=C_{3z}^{2}P_{2}^{\pm }$.

**Fig. 2.**
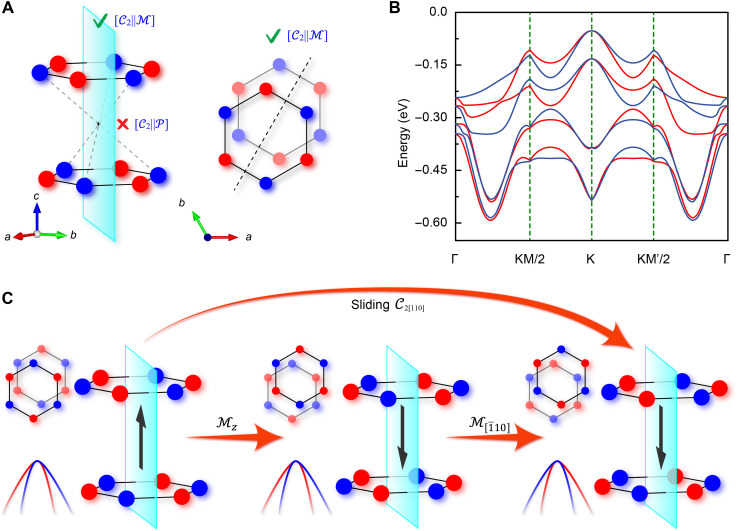
Impact of polarization switching on altermagnetic band structure. (**A**) Magnetic ordering configuration of bilayer MnPS_3_, where red and blue represent spin-up and spin-down Mn lattice sites, respectively. (**B**) Energy band structure with altermagnetic characteristics. (**C**) Decomposition of the spatial symmetry operations corresponding to the polarization switching achieved by layer sliding. The black arrow represents the out-of-plane polarization direction, and the cyan plane indicates the mirror symmetry. The inset on the left shows a top view of the magnetic sites and a schematic of the spin splitting direction, indicating that the momentum space spin splitting remains unchanged under $M_{z}$ but is reversed under $M_{\left[\bar{1}10\right]}$.

Within the framework of spin-space group theory, altermagnetism requires that opposite-spin sublattices be coupled through rotational or mirror (M) symmetries but not through translational or inversion symmetries—since the latter would cause the system to degenerate into a conventional antiferromagnet (*29*, *30*). Figure 2A shows the ground-state magnetic configuration of bilayer MnPS_3_ in the $P_{3}^{+}$ state (energy comparisons with other magnetic configurations are provided in fig. S5). Here, the inherent lack of translational symmetry in the hexagonal lattice, combined with the P symmetry breaking induced by sliding ferroelectricity, creates the necessary symmetry conditions. Crucially, the mirror symmetry of the system forbids any component of the polarization vector that is perpendicular to the mirror plane. As a result, the in-plane component of the sliding ferroelectric polarization is rigidly locked to the mirror plane direction, imposing an altermagnetism constrained by the $\left[C_{2}\|M_{\left[\bar{1}10\right]}\right]$ symmetry, as illustrated in Fig. 2B. The complete band structure, including the conduction bands, is shown in fig. S6. On the basis of this symmetry analysis, the transition from the $P_{3}^{+}$ to the $P_{3}^{-}$ state (path 1) is this by the $C_{2\left[110\right]}$ operation. This symmetry operation is equivalent to the composite operation $M_{z}M_{\left[\bar{1}10\right]}$, where the former maps the lattice polarization structure and reverses the polarization, while preserving the two-dimensional (2D) momentum space because $M_{z}\left(k_{x},k_{y}\right)=\left(k_{x},k_{y}\right)$, and the latter restores the original spin configuration, as shown in Fig. 2C. This combination ensures the consistency of spin configurations between the two polarization states. When combined with the altermagnetic $\left[C_{2}\|M_{\left[\bar{1}10\right]}\right]$ symmetry, the polarization switching transforms the energy eigenstate as follows

$$C_{2\left[110\right]}E\left(s,k\right)=M_{z}M_{\left[\bar{1}10\right]}\left[C_{2}\|M_{\left[\bar{1}10\right]}\right]E\left(s,k\right)=E\left(-s,k\right)$$ (1)

Furthermore, collinear magnets have a fundamental symmetry $[{\bar{C}}_{2}||T]E\left(s,k\right)=E\left(s,-k\right)$, which implies

$$C_{2\left[110\right]}E\left(s,k\right)=[C_{2}||T]E\left(-s,k\right)=E\left(-s,-k\right)=TE\left(s,k\right)$$ (2)

This result indicates that the purely spatial operation of ferroelectric reversal is equivalent to a pseudotime-reversal effect, which preserves the magnetic configuration in real space while applying an effective time-reversal operation T to the energy eigenstates in momentum space, thereby locking the direction of the ferroelectric polarization with that of the spin polarization, as illustrated in Fig. 2C.

**Fig. 3.**
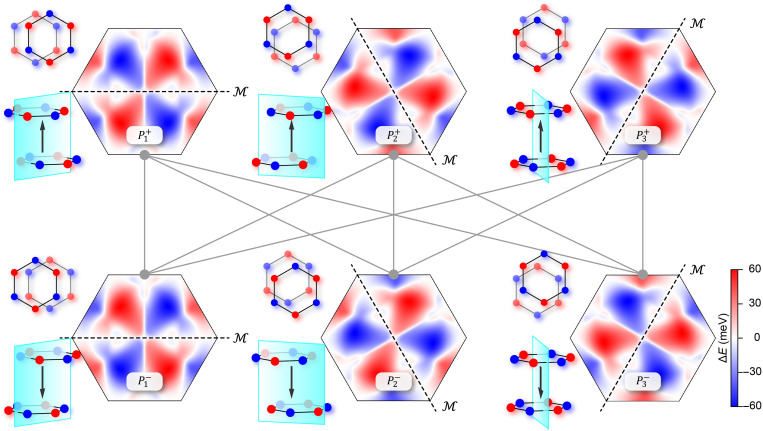
Spin-splitting textures in the first Brillouin zone for six different polarization states and their corresponding structural stacking configurations. The dashed lines and the cyan plane mark the mirror direction of the system, which rigidly aligns with the in-plane polarization direction, revealing six distinct momentum-space spin polarization reconstructions controlled by polarization.

Therefore, the multistate polarization enabled by sliding ferroelectricity offers a distinct advantage for controlling altermagnetism: Polarization switching not only couples strongly to the direction of spin splitting but also allows deterministic reversal of the in-plane polarization, equivalent to applying crystal rotations of 0°, 120°, or 240°. This mechanism directly enables sixfold nonvolatile magnetoelectric coupling within a simple bilayer system. As shown in Fig. 3, the spin-splitting textures across all six polarization states unambiguously reveal the *d*-wave symmetric altermagnetism. Their corresponding band structures are shown in fig. S7. In particular, as shown in the inset of Fig. 3, mirror symmetry inherently locks the in-plane polarization while simultaneously governing the system’s altermagnetism. As a result, electric field–driven polarization switching permits precise and deterministic manipulation of the spatial distribution and anisotropy of the altermagnetic spin splitting. This constitutes a universal symmetry-locking mechanism, establishing a paradigm-defining framework for high-dimensional magnetoelectric coupling and making it applicable to systems with the same spin-space symmetry, such as bilayer MnPX_3_ and VPX_3_ (X = S, Se, Te) with Néel-type magnetic ordering.

Nonrelativistic spin polarization in altermagnets provides a unique platform for exploring quantum transport phenomena (*31*–*37*). In particular, their distinctive magnetic geometry induces a pronounced BCD, driving an intrinsic NLH effect (*38*). Leveraging the six-state electrical control of spin polarization’s spatial distribution, the six polarization directions ($P_{1}^{\pm }$, $P_{2}^{\pm }$, and $P_{3}^{\pm }$) correspond to distinctly different crystallographic symmetrical environments. This characteristic precisely matches the inherent sensitivity of the BCD to lattice anisotropy, resulting in distinctly discrete BCD distributions, and further enables efficient and deterministic electrical switching between six distinct NLH responses.

In general, the nonlinear conductivity tensor is defined as the quadratic current response **J** to current-driving electric field **E**

$$J^{\alpha }=\chi^{\text{αβγ}}E^{\beta }E^{\gamma }$$ (3)

Among these, the nonlinear conductivity tensor $\chi^{\text{αβγ}}$ stems from the BCD tensor *D* contribution (*39*, *40*)

$$\chi^{\text{αβγ}}=\epsilon^{\text{αδγ}}D^{\text{βδ}}$$ (4)

$$D^{\text{βδ}}=\frac{e^{3}}{\hbar^{2}\tau }\int \left[dk\right]f_{nk}{\text{∂}}_{\beta }\Omega_{\delta }$$ (5)

where [*d***k**] is the short notation of $\sum_{n}dk/{\left(2\pi \right)}^{2}$, *n* is the band index, *f*_*n***k**_ is the Fermi-Dirac distribution function, and Ω is the Berry curvature tensor. In 2D systems, the Berry curvature reduces to a pseudoscalar, with only the *z* component being nonvanishing. Consequently, the nonzero components of the BCD tensor $D^{\beta \delta }$ must satisfy δ = *z*, yielding two electrically tunable elements: $D^{\mathrm{xz}}$ and $D^{\mathrm{yz}}$. These correspond directly to the measurable NLH conductivities $\chi^{\mathrm{yxx}}$ and $\chi^{\mathrm{xyy}}$, which can be extracted by applying an in-plane current-driving electric field $E^{x}$ or $E^{y}$ and measuring the transverse second-harmonic voltage signal.

**Fig. 4.**
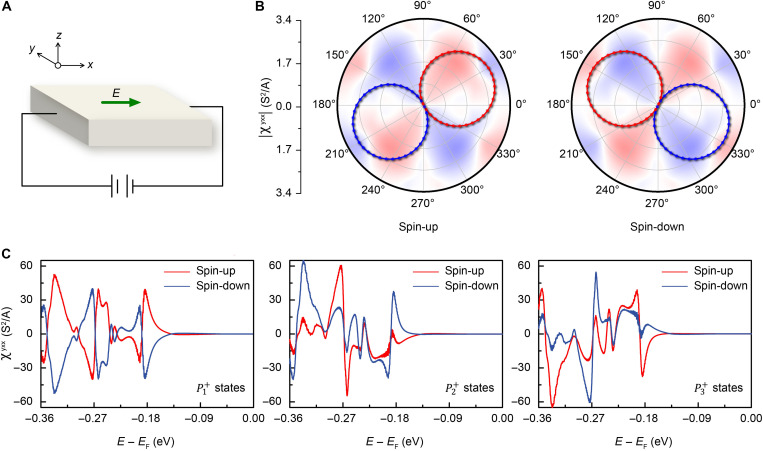
Angle dependence of NLH effect. (**A**) Schematic of the NLH effect generation mechanism under an electric field along the *x* direction. (**B**) Angular dependence of the conductivity $\chi^{\mathrm{yxx}}$ in different spin channels, along with the corresponding spin splitting geometry in the first Brillouin zone. The 2π-periodic sine relationship reflects the high sensitivity of the NLH response to magnetic geometry. The red and blue lines represent positive and negative values, respectively. (**C**) NLH conductivity $\chi^{\mathrm{yxx}}$ contributed by BCD in the $P_{1}^{+}$, $P_{2}^{+}$, and $P_{3}^{+}$ states.

As illustrated in Fig. 4A, when an electric field $E^{x}$ is applied, the NLH conductivity $\chi^{\mathrm{yxx}}$ exhibits distinct values for polarization states $P_{1}^{+}$, $P_{2}^{+}$, and $P_{3}^{+}$ (Fig. 4C). For $P_{1}^{+}$ state, the lattice mirror is parallel to the *x* direction, which aligns with the current-driving electric field $E^{x}$. This results in opposite spin channels contributing exactly opposite NLH, with a peak intensity of 36.4 S^2^/A at approximately 0.18 eV, which is within the range of typical measurable intensities using existing NLH effect measurement techniques. For $P_{2}^{+}$ and $P_{3}^{+}$ state, the lattice mirror forms a 120° angle with the direction of the current-driving electric field, leading to asymmetric contributions to the NLH from the opposite channels. Specifically, in the $P_{2}^{+}$ state, the spin-down channel contributes a strong positive peak of 36.9 S^2^/A at 0.18 eV, while the spin-up channel shows a strongly suppressed weak negative value of 1.2 S^2^/A. In the $P_{3}^{+}$ state, the spin-up and spin-down channels contribute a weak positive value and a strong negative value, respectively, with intensities that are exactly the same as those observed in the $P_{2}^{+}$ state, as shown in Fig. 4C. To further elucidate the angular dependence of the NLH conductivity on magnetic geometry, we calculated $\chi^{\mathrm{yxx}}$ in the *xy* plane for $P_{1}^{+}$ state at −0.18 eV. Our results reveal a periodic sinusoidal angular dependence of $\chi^{\mathrm{yxx}}$ with a period of 2π, confirming its high sensitivity to magnetic geometry and lattice anisotropy, as shown in Fig. 4B.

**Fig. 5.**
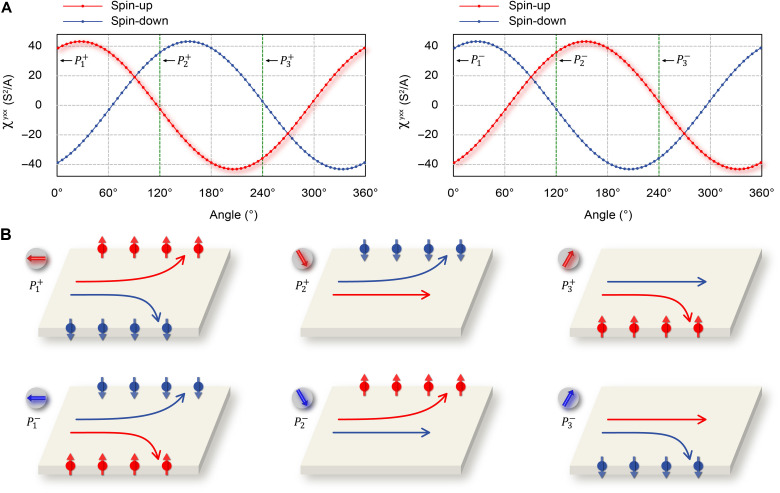
NLH conductivity behavior in six different ferroelectric states. (**A**) The NLH conductivity as a function of angle, with $P_{1}^{\pm }$, $P_{2}^{\pm }$, and $P_{3}^{\pm }$ corresponding to 0°, 120°, and 240°, respectively. In these states, the spin-up and spin-down channels are fully swapped. This behavior arises from the polarization switching induced by pseudotime-reversal symmetry $C_{2}E\left(s,k\right)=TE\left(s,k\right)$. (**B**) Schematic of the six distinct corresponding NLH signals mediated by sliding ferroelectrics.

Polarization switching intrinsically reverses the out-of-plane component—a process equivalent to a pseudotime-reversal operation that exchanges spin-up and spin-down channels via $C_{2}E\left(s,k\right)=TE\left(s,k\right)$. Figure 5A demonstrate the angular dependence of the NLH conductivity $\chi^{\mathrm{yxx}}$ at −0.18 eV. According to $P_{3}^{\pm }=C_{3z}^{1}P_{1}^{\pm }=C_{3z}^{2}P_{2}^{\pm }$, the $P_{1}^{\pm }$, $P_{2}^{\pm }$, and $P_{2}^{\pm }$ states correspond to specific angles of 0°, 120°, and 240°, respectively. The influence of polarization switching on the NLH conductivity is shown in fig. S8. We thus achieve dual control: In-plane polarization governs NLH current deflection, while out-of-plane polarization dictates spin-channel exchange. Figure 5B summarizes the six distinct transport regimes, revealing mutually reversed current deflection in opposite spin channels for $P_{1}^{\pm }$ states, whereas $P_{2}^{\pm }$ and $P_{3}^{\pm }$ configurations exhibit strong suppression in one spin channel alongside spin-polarized deflection in the other. Therefore, by switching the sliding ferroelectric polarization, it is possible to achieve nonvolatile, deterministic switching between six distinct NLH signals in a simple bilayer system, offering strong potential for applications in multistate memory, spin-based logic, and reconfigurable neural network hardware.

## DISCUSSION

In this study, we have demonstrated deterministic electrical control over multiple altermagnetic degrees of freedom through sliding ferroelectricity—a symmetry engineering strategy that creates a rigid coupling between polarization direction and spin-splitting geometry. Specifically, the in-plane polarization is locked to crystal mirror symmetry, while the out-of-plane components governs spin-channel exchange via a pseudotime-reversal operation. This dual mechanism allows polarization switching not only to reverse the spin splitting but also to systematically rotate its spatial anisotropy in increments of 120°, resulting in six nonvolatile altermagnetic states in the bilayer MnPS_3_ system. Crucially, each state generates a distinct NLH response mediated by the BCD, underscoring the functional viability of our multistate platform.

Compared to the traditional binary control paradigm, the six-state control achieved in this study offers clear advantages. In neuromorphic computing, the six distinct and stable magnetic states provide a more refined simulation of the higher-order connections and complex states found in biological synapses, offering a richer physical basis for efficiently simulating learning and memory processes. In information storage, the six-state mechanism not only enables higher data density but also facilitates faster data retrieval and lower energy consumption by reducing the need for frequent state switching. The multistate, nonvolatile, and electrically controllable nature of this system allows a single device to support more complex logic and storage functions, providing a highly promising platform for building next-generation information hardware with high parallelism and integrated computation and storage capabilities.

## METHODS

The atomic properties and electronic structure of the materials were calculated using first-principles simulations within density functional theory (DFT) (*41*, *42*). The projected augmented wave pseudopotentials method, as implemented in the Vienna Ab initio Simulation Package (*43*, *44*) was used. The exchange-correlation energy was calculated using the generalized gradient approximation of the Perdew-Burke-Ernzerhof form (*45*), and the plane wave cutoff energy was set to 500 eV. A Hubbard *U*_eff_ = 4 eV with the Dudarev parametrization was applied to properly describe the localization of Mn 3*d* orbitals (*46*). The semiempirical DFT-D3 method was used to include the van der Waals interaction (*47*). For MnPS_3_ calculations, a centered 9 × 9 × 1 Monkhorst-Pack *k*-point mesh was used (*48*). To eliminate periodic boundary effects, the vacuum space between adjacent slabs was set to exceed 15 Å along the *z* direction. The atomic relaxation and subsequent electronic structure processing and polarization calculations were carried out using a unified convergence criterion, with energy and force converging to 10^−6^ and 10^−2^ eV/Å, respectively. The Berry-phase method was used to evaluate polarization magnitude (*49*), and the ferroelectric transition switching pathway was obtained using the climbing image nudged elastic band method, with energy and force converging to 10^−5^ and 2 × 10^−2^ eV/Å, respectively (*50*). Tight-binding models are constructed from DFT bands using the WANNIER90 package (*51*), and within the WannierBerri code (*39*), the BCD tensors are calculated in their gauge-covariant form (*52*), with a relaxation time set τ = 1 ns.
