# Supplementary material for: Symmetry-locked six-state control of altermagnetism via sliding ferroelectricity
Source: Sci Adv. 2026 Jun 3;12(23):eaec5229. doi: 10.1126/sciadv.aec5229 (PMC13232588; doi:10.1126/sciadv.aec5229)
Supplement: Supplementary file 1 — Figs. S1 to S8 [file sciadv.aec5229_sm.pdf]

Science Advances
AAAS

Supplementary Materials for

Symmetry-locked six-state control of altermagnetism via sliding ferroelectricity

Wei Sun *et al.*

Corresponding author: Wenxuan Wang, mse_wangwx@ujn.edu.cn; Shifeng Huang, mse_huangsf@ujn.edu.cn; Zhenxiang Cheng, cheng@uow.edu.au

Sci. Adv. **12**, eaec5229 (2026)
DOI: 10.1126/sciadv.aec5229

This PDF file includes:

Figs. S1 to S8

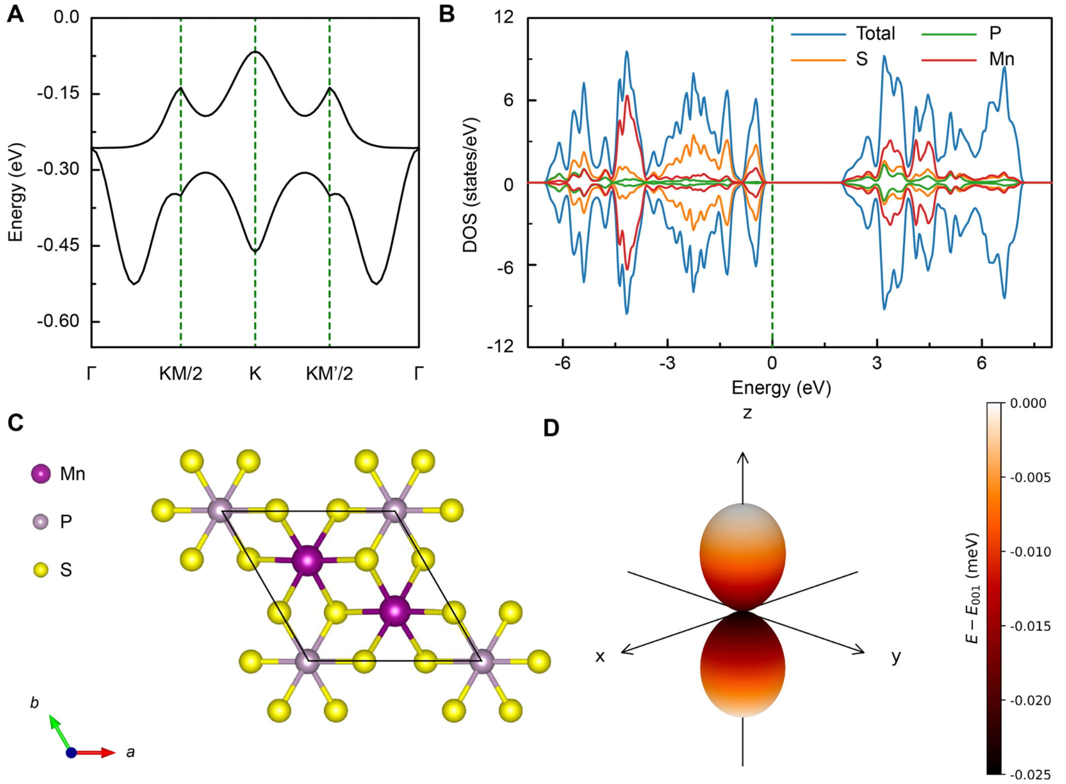

Fig. S1. Electronic and magnetic properties of monolayer $MnPS_3$. (A) and (B) represent the band structure and density of states of monolayer $MnPS_3$, respectively. The spin-degenerate band structure clearly demonstrates that monolayer $MnPS_3$ is a conventional antiferromagnetic semiconductor. (C) presents the top view of the monolayer $MnPS_3$ structure with a lattice constant of 6.046 Å. (d) displays the magnetic anisotropy energy, indicating easy-plane anisotropy.

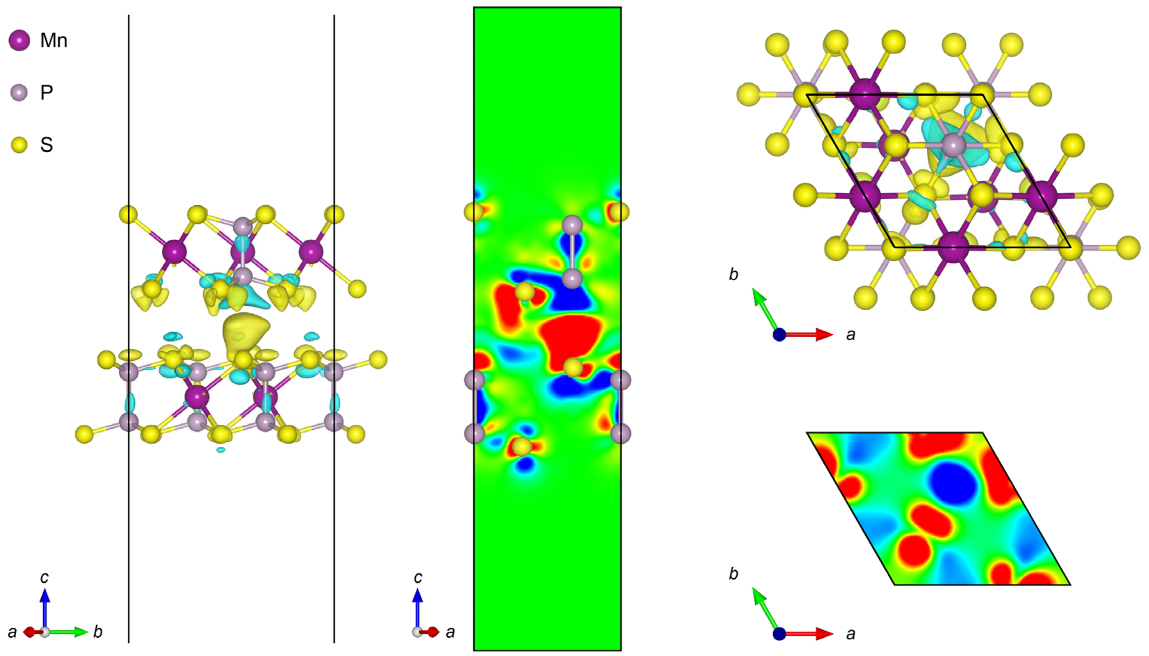

Fig. S2. Differential charge distribution of bilayer $MnPS_3$. The side and top views of the

differential charge spatial distribution, along with its cross-sections on the $(\bar{1}10)$ and (001) crystallographic planes. The out-of-plane electric dipole moment originates from the accumulation of charge at the interface S atoms, while it is depleted at the P atoms. This charge redistribution is attributed to the difference in electronegativity between S and P atoms. Additionally, the in-plane charge transfer follows a mirror-symmetric distribution with respect to the $(\bar{1}10)$ plane, generating an in-plane dipole moment along the [110] direction.

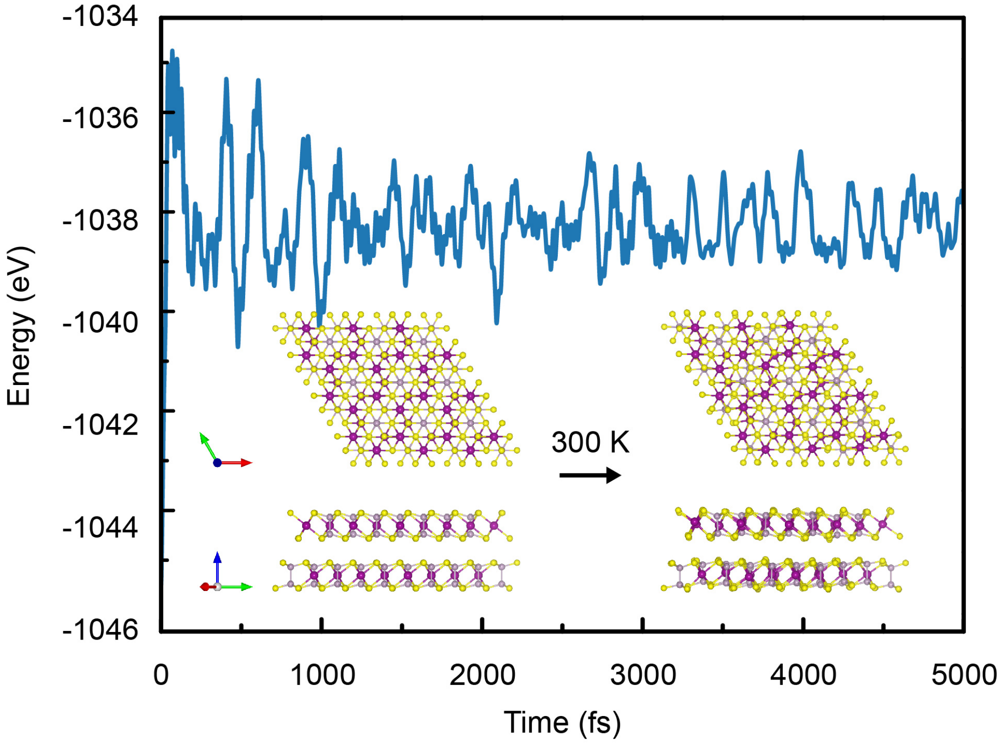

Fig. S3. ***Ab*** **initio molecular dynamics (AIMD) simulation of bilayer $MnPS_3$.** The simulation was performed on a 3×3×1 supercell, using the NVT ensemble with a Nosé-Hoover thermostat to model thermal effects. The total potential energy as a function of AIMD time for 5 ps with a time step of 1fs at 300 K. The inset shows snapshots at 0 ps and 5 ps.

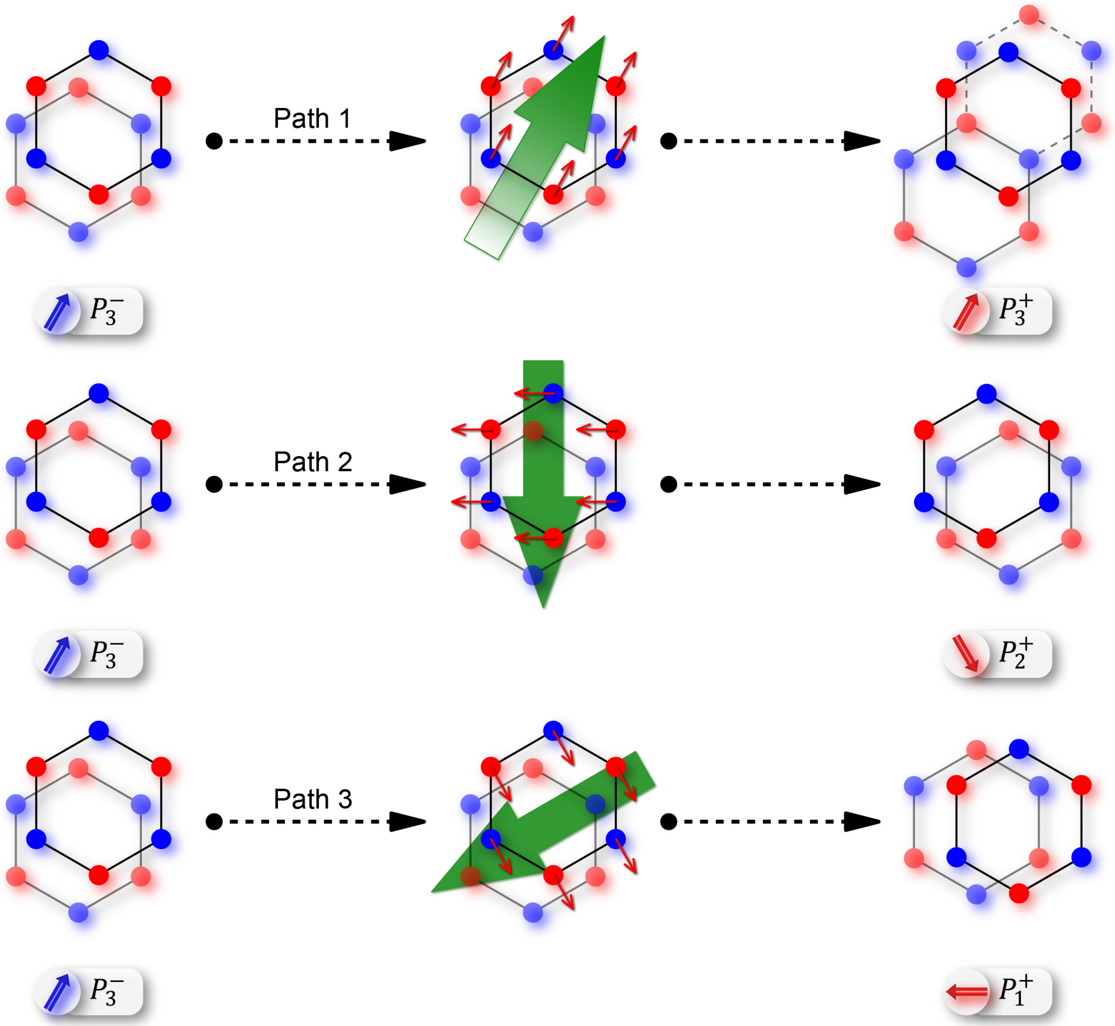

Fig. S4. Controllable switching of the six polarization states achieved by the external electric field. The green arrows represent the direction of the applied electric field, where for Path 1, a combination of in-plane and out-of-plane fields is required, while only an in-plane electric field is needed for Paths 2 and 3. The red arrows indicate the direction of ionic displacement required to complete the polarization switching.

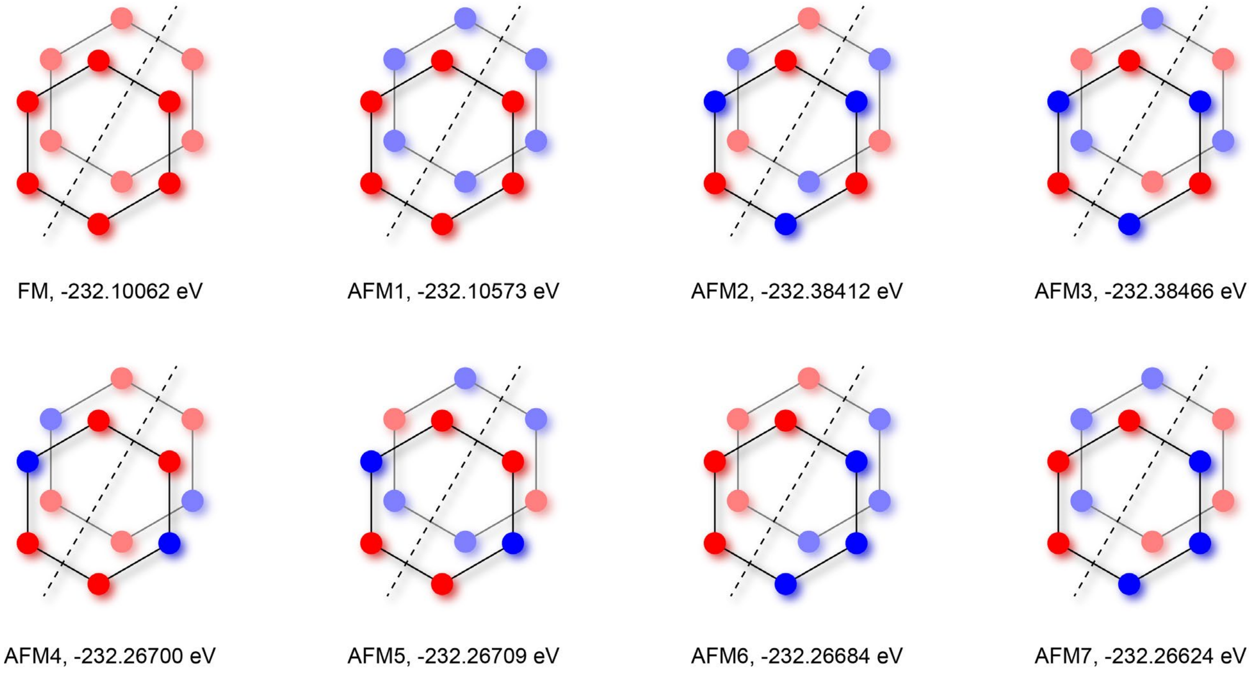

Fig. S5. The total energy of the $MnPS_3$ bilayer under different magnetic orderings. A total of one ferromagnetic and seven antiferromagnetic configurations were considered, among which AFM3 has the lowest energy and corresponds to the ground-state magnetic ordering of the system. Red and blue represent opposite spins, and the dashed line indicates the mirror symmetry of the $MnPS_3$ bilayer.

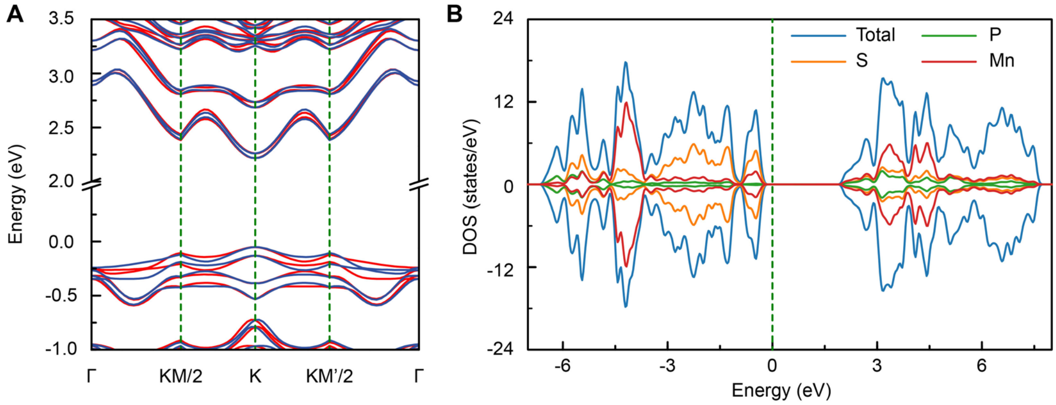

Fig. S6. The electronic structure of $MnPS_3$ bilayer. (A) and (B) represent the band structure and density of states of $MnPS_3$ bilayer, respectively. The spin-splitting band structure reveals its semiconducting nature with altermagnetic characteristics and a band gap of 2.27 eV.

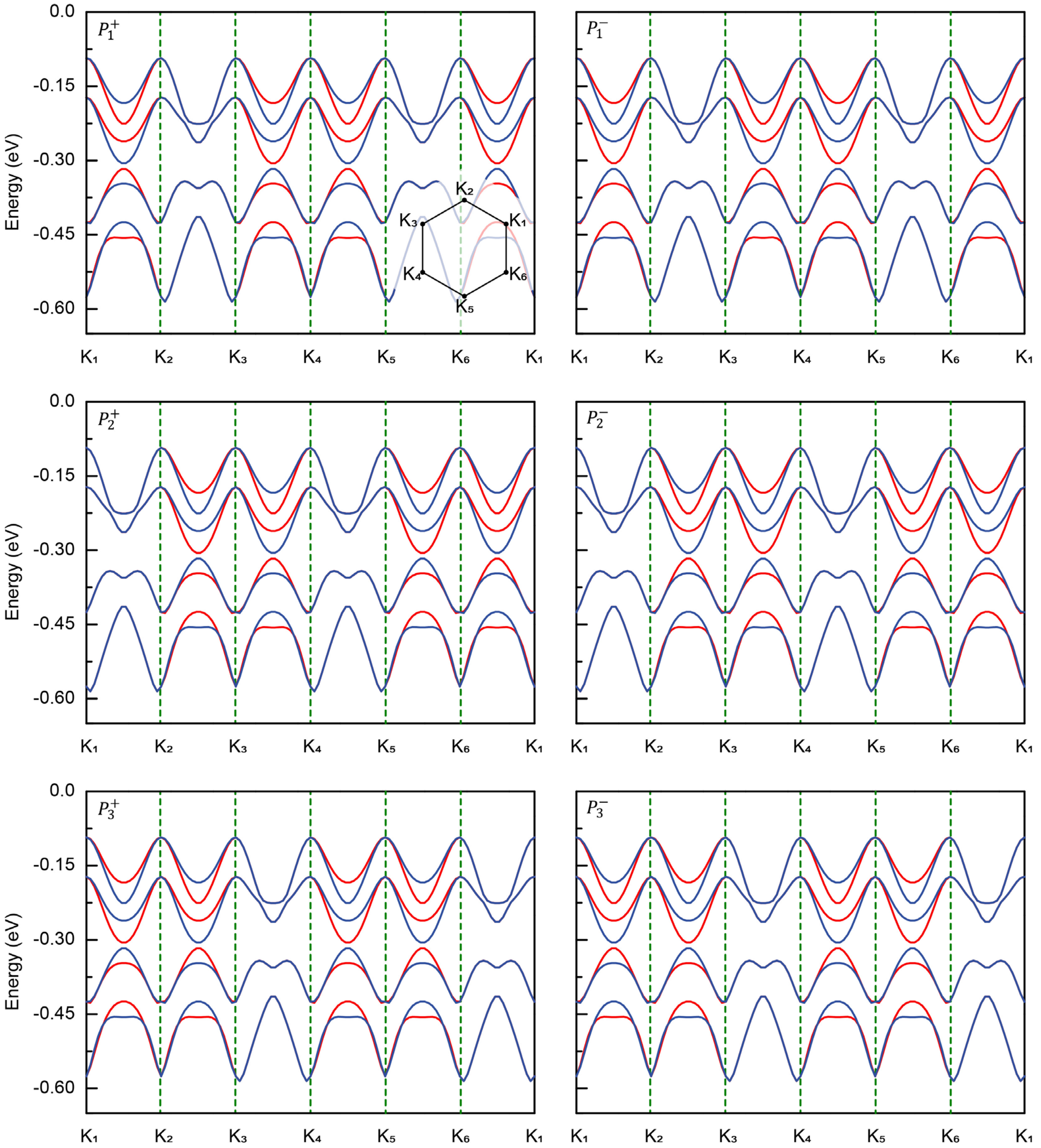

Fig. S7. Band structures corresponding to six different polarization states. In-plane polarization controls the spin splitting to occur along different high-symmetry paths, while switching the out-of-plane component reverses the direction of spin splitting. Thus, the spin-splitting paths and their directions vary across these six polarization states.

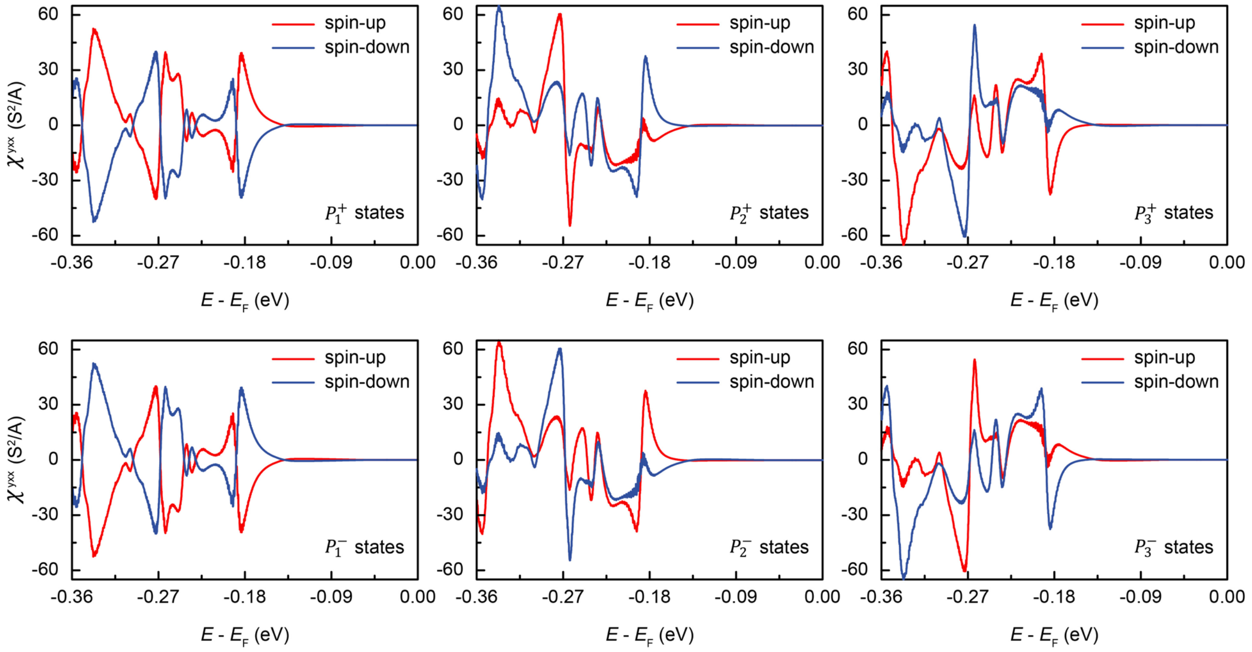

Fig. S8. NLH conductivity χ^{yxx} in six different polarization states. The spin components of the NLH conductivity are exactly opposite for the P_n^+ and P_n^- states, where n = 1, 2, 3.
